# A qualitative exploration of factors that influence the uptake of tuberculosis services by low-skilled migrant workers in Singapore

**DOI:** 10.1186/s12913-023-09938-y

**Published:** 2023-09-02

**Authors:** Chuan De Foo, Shishi Wu, Fariha Amin, Natarajan Rajaraman, Alex R. Cook, Helena Legido-Quigley

**Affiliations:** 1 Saw Swee Hock School of Public Health, National University of Singapore and National University Health System, Singapore, Singapore; 2 Duke-NUS Graduate Medical School, Singapore, Singapore; 3 Dalla Lana School of Public Health, University of Toronto, Toronto, ON Canada; 4 HealthServe Community Clinic, Singapore, Singapore; 5 Maluk Timor, Dili, Timor Leste Timor-Leste; 6 Imperial College and the George Institute for Global Health, London, UK

**Keywords:** Singapore, Tuberculosis, Qualitative study, Migrant workers

## Abstract

**Introduction:**

Singapore relies heavily on migrant workers to build its country and harbours a relatively large population of these workers. Importantly, tuberculosis (TB) remains a pernicious threat to the health of these workers and in line with the United Nations High-Level Meeting in 2023, this paper aims to uncover the qualitative discourse facing migrant workers’ uptake of TB services and provide policy recommendations to enable more equitable access to TB services for this population.

**Methods:**

In-depth interviews were carried out with the migrant worker population recruited from a non-governmental organisation in Singapore that serves migrant workers through the provision of primary healthcare services, counselling, and social assistance. Interviews stopped once thematic saturation was achieved and no new themes and subthemes were found.

**Results:**

A total of 29 participants were interviewed, including 16 Bangladeshis and 13 Chinese, aged between 22 and 54 years old, all worked in the construction sector. Four key themes emerged. They are (1) General TB knowledge: Misconceptions are prevalent, where we found that participants were aware of the disease but did not possess a clear understanding of its pathophysiology and associated health effects, (2) Contextual knowledge and perception of associated policies related to TB in Singapore: low awareness among migrant workers as participants’ accounts depicted a lack of information sources in Singapore especially on issues related to healthcare including TB, (3) Attitude to towards TB: Motivation to seek treatment is underpinned by ability to continue working and (4) Stigma: mixed perception of how society views TB patients. The gaps identified in migrant workers’ TB knowledge, their attitude towards the disease and their perception of the availability of TB-related services is despite Singapore’s efforts to curb community spread of TB and its proactive initiatives to reduce the prevalence.

**Conclusion:**

Our study illuminates the various aspects that policymakers need to home in on to ensure this vulnerable group is sufficiently supported and equitably cared for if they develop active TB during their stay in Singapore as they contribute to the nation’s economy. Leveraging the COVID-19 pandemic as a window of opportunity to improve overall healthcare access for vulnerable groups in Singapore can be a starting point.

**Supplementary Information:**

The online version contains supplementary material available at 10.1186/s12913-023-09938-y.

## Introduction

Tuberculosis (TB) is one of the world’s deadliest communicable diseases. The World Health Organization (WHO) reports that approximately 10 million people developed TB in 2017, of whom more than half (62%) were in the South-East Asia and Western Pacific Regions [1]. Following a series of intervention schemes, the incidence of TB in Singapore has declined from 307 cases per 100,000 in 1960 to an all-time low of 35 cases per 100,000 in 2007. Since 2007, there has been a resurgence of TB in Singapore. However, the incidence hovered in the range of 49 to 57 cases per 100,000 for the following 10 years. In 2017, a total of 2,191 new TB cases were notified in Singapore, 655 (30%) of them were long-staying foreigners [2]. Among them, 446 newly reported cases were migrant workers^1^, making the incidence of TB among them around 46 cases per 100,000 population [2, 3].

Recently, much focus has also been placed on latent TB infection (LTBI), a *Mycobacterium tuberculosis* infection without the clinical manifestation of active TB [4]. It was estimated that 1.7 billion people or 23% of the world’s population had LTBI in 2014 [5], and relatively high TB incidence countries such as Singapore are encouraged to strategically identify high-risk groups for LTBI and provide necessary treatment to reduce the probability of progression to active TB [4]. One of the high-risk groups is immigrants entering Singapore from other high TB burden countries [4, 6]. Most migrant workers in Singapore come from Bangladesh, China, India, Indonesia, Myanmar, Thailand and the Philippines, which are found on the WHO’s lists of high-burden TB countries [1]. In Singapore, all migrant workers require a medical examination that screens for four types of infectious diseases, including active TB, before they are given a work permit [7]. While this measure prevents applicants with active TB from obtaining work permits, migrants with LTBI continue to obtain permits to work in Singapore or may develop TB during their employment in the country and programmes may be needed to ensure they receive adequate care. Hence, a closer look into the programmatic management of LTBI is essential to stem the progression to and spread of active TB among migrant workers in Singapore.

As of December 2022, there were 1.42 million non-residents in Singapore, among whom more than half were low- or semi-skilled migrant workers [3]. In recent years, migrant’s health needs have been increasingly emphasised in the international policy agenda. The COVID-19 pandemic further shone the spotlight on this vulnerable population, which has been seen to suffer disproportionately during the health crisis. The same dense living conditions that predispose them to getting infected by SARS-CoV-2 also put them at risk of acquiring *Mycobacterium tuberculosis* infections. There are biological and social interactions between TB and COVID-19, whereby their interplay increases the likelihood of contracting one or the other [8]. Such scenarios play out in low- and middle-income countries (LMICs) but, surprisingly, are also highly pertinent to high-income countries such as Singapore, with the COVID-19 pandemic displaying inequitable health services delivery and lack of person-centred care for its low-skilled migrant worker population.

The need to tackle the inequities surrounding healthcare access for this population is critical, especially since migrant labour is a crucial component for various economic activities. Yet, migrants’ access to fundamental human rights and other essential services is often compromised by the lack of effective legislation and enforcement. In general, migrants are more prone to poor health, to engage in unhealthy behaviours, and to have poor access to healthcare [9–12]. One of the significant initial steps in curbing TB and LTBI among migrant workers is to understand their knowledge, attitudes and behaviours pertaining to the disease, which will impact the uptake of healthcare services that manage TB in this vulnerable population. A systematic review synthesising qualitative evidence on migrants’ knowledge and attitudes suggested that migrants had little knowledge about TB and that widespread misconceptions about TB are not uncommon. Migrants also generally delay seeking treatment despite experiencing symptoms for a extended periods of time [13]. To date, the literature on migrant workers’ health literacy and health-seeking behaviours in Singapore is scarce; let alone the much-needed attention on TB and its associated qualitative discourses. Importantly, most studies on TB and affiliated programmes are for vulnerable groups in LMICs [14–16]. Singapore, a high-income country, despite having a relatively large population of migrant workers, does not have robust qualitative studies on the uptake of such services and this population’s willingness to take up TB-related services. The paucity of evidence impedes efforts to advocate for equitable access to a full continuum of health services spanning primary prevention, diagnosis and treatment. Therefore, this research aims to address this gap by offering a qualitative discourse on migrant workers’ uptake of TB-related services in Singapore. Importantly, this work is also in line with the upcoming United Nations High-Level Meeting (UN HLM) in September 2023, where stop TB priorities are reaffirmed internationally [17]. As priority shifts following various systematic shocks in recent years such as from climate change afflictions, the war in Ukraine and the ongoing COVID-19 pandemic, a firm endorsement to bring an end to the TB epidemic in all populations in Singapore ought to remain at the top of the agenda.

## Methods

Recruitment of participants was done in collaboration with HealthServe, a Non-Governmental Organisation (NGO) in Singapore that served migrant workers through the provision of primary health services, counselling, and social assistance [18]. Staff and volunteers of HealthServe facilitated recruitment of prospective participants. Migrants who visited HealthServe Clinic in the Geylang district and spoke Bengali or Chinese were approached for recruitment. Recruitment ceased when thematic saturation was achieved, such that subsequent interviews were unlikely to lead to new information. The researchers who conducted the interviews were trained qualitative researchers experienced in working with vulnerable migrant populations. Written informed consent in a language that the migrants understood was obtained prior to interviews, permission to be audio-recorded and quoted anonymously in research outputs was also obtained. All interviews were conducted in private rooms arranged by HealthServe to ensure confidentiality. Migrants were reassured that all information would be kept confidential and that their participation would not affect the services they received from HealthServe clinics. Participants were also reminded that they could withdraw their participation at any time. Ethical approval was obtained from the National University of Singapore Institutional Review Board (NUS-IRB: S-18-197).

Semi-structured in-depth interviews guided by a topic guide were conducted face-to-face with twenty-nine semi-skilled migrants who hold work permits in Singapore. In-depth interviews allow participants to freely discuss their experiences and perceptions about TB without losing focus. Broadly, the participants were first asked a series of questions pertaining to the research question regarding the TB and TB-relevant services in Singapore. When the trained interviewer felt that there were more perspectives to be shared by the participant, the interviewer probed the participant to elicit more information. This is done in a semi-structured format to ensure a comprehensive and in-depth understanding of the migrant workers’ outlook and factors affecting TB and TB services uptake. Operationally, the interviews were conducted in Bengali and Chinese Mandarin and the sessions were also audio-recorded. Each interview lasted an average of 45 min.

Interviews were transcribed and translated verbatim into English simultaneously by professional transcriptionists. The research team proofread all transcripts before data analysis to ensure that the transcription and translation were done literally without losing accurate meanings. QSR NVivo 12 software was used to manage and organise the data. Data generated from the interviews were approached through an interpretivist lens to focus on participant’s experiences, perceptions and how they understand and reason with the topic discussed. Two authors first coded half of the transcripts each, line-by-line and inductively [19]. After the initial coding was completed, the two authors compared the two sets of codes to ensure inter-coder reliability and to allow for data triangulation. The coders were engaged in regular discussions to identify emerging themes and seek out deviant cases. In the meantime, recruitment and interviews continued in parallel, followed by audio transcription and thematic analysis. These collection, consolidation and analysis steps only stopped when thematic saturation was reached and no new themes emerged. Disagreements were resolved by discussing and consulting with a third researcher who provided neutral views on the analysed data set. Eventually, a coding frame was agreed to and then used to re-code the whole set of data to ensure consistency. All authors held regular debriefings on the pre-conceived ideas and perceptions throughout the study period to ensure as much neutrality as possible.

The Standards for Reporting Qualitative Research (SRQR) Checklist was used to guide thequalitative research methodology. The SRQR checklist is included as a supplementary material.

## Results

### Demographic information

A total of 29 participants were interviewed, including 16 Bangladeshis and 13 Chinese, aged between 22 and 54 years old, all worked in the construction sector. Most were married with one or two children. About half of the participants had worked in Singapore for more than 10 years. Participants’ accommodation was provided by the employers, but most reported sharing a room with more than 10 other migrant workers (10 to 30 roommates depending on room sizes). Participants also shared that they worked 8 to 16 h on a typical workday, with a statutory off day on Sunday.

For the aim of this paper, we organised our findings according to four main themes: general TB knowledge, contextual knowledge and perceptions related to TB and associated policies in Singapore, attitude towards TB, and stigma related to TB.

### Main theme 1. General TB knowledge: Misconceptions are prevalent

All participants were aware of the disease but did not possess a clear understanding of its pathophysiology and associated health effects. Many could only give an ambiguous explanation of what they knew or thought they knew about TB. Participants’ knowledge is further categorised into the subthemes of “Perception of TB disease”, “Causes of TB”, “TB prevention and treatment”, “Sources of TB information”.

### Subtheme 1.1: Perception of TB disease

Most participants understood TB as pulmonary TB, resulting in them sharing symptoms affecting the respiratory system, such as *“coughing a lot”* or *“coughing with blood”*. Some participants associated TB with flu-like symptoms (such as fever, sore throat, and chills) or respiratory problems (such as choking and shortness of breath). Other responses the participants gave included shadows on lung X-rays, sleeping problems and nausea. Few participants had heard about “carrier” (latent TB) being non-contagious and none of the participants had heard of multidrug-resistant TB (MDR-TB).

### Subtheme 1.2: Causes of TB

Participants tend to generalise the transmission of TB into other infectious diseases, and for many participants, different causes of TB sometimes co-exist. Bad hygiene, airborne infection, weak immunity, contact with patients and food sharing are among the most frequently cited causes of TB. In addition, while a small number of participants thought that TB is a progression from chronic coughs and two participants thought that TB is hereditary, many participants believed that smoking is a major cause of TB. Other potential causes of TB shared by participants included kitchen smoke, bad odour in the environment, drinking alcohol and using narcotics, being in a cold environment, and exchanging bodily fluids with TB patients.

*“In a damp environment, like if the place is too muddy, if the place is not clean. As you know a lot of times when smoke from kitchen infiltrates the living space […] Sometimes people have domestic cows (back in their home country). When people live with cows, they have to live with odour from cows. All these can cause tuberculosis. And those who smoke cigarettes, they can catch TB from cigarettes.” (M04-B)*.

### Subtheme 1.3: TB prevention and treatment

Most participants shared that they did not know how to prevent TB and some believed TB prevention is beyond their control. Among those who knew, measures such as quitting smoking and avoiding sharing utensils with TB patients were frequently mentioned as essential in TB prevention. Other responses included avoiding dust, keeping a distance from TB patients, doing regular physical activities, quitting alcohol and ensuring a hygienic environment. A few participants believed they could prevent TB by avoiding cold drinks, burying TB patients’ sputum, and using home remedies to prevent colds.

While some participants doubted if TB is curable, most believed that treatment is available for TB and that a cure is possible. However, they usually could not elaborate on the mode or length of treatment.

Acknowledging their lack of understanding about TB, most participants attributed it to not having experienced or witnessed it themselves, which can be demonstrated by the following quote:

*“Because I never got to see anyone in my family or relatives suffering from this disease (TB). That’s why I don’t know anything about this.” (M13-B)*.

A small number of participants who witnessed relatives having TB could share more about the symptoms they had observed but could not elaborate on the details of transmission and treatment.

### Subtheme 1.4: Sources of Information

The low level of TB-related knowledge among the participants could be attributed to the lack of access to relevant information from reliable sources. The primary source of information, as shared by participants, was advertisements on television. Some participants knew about TB through witnessing TB cases among their relatives or friends back home or heard about it through friends. A handful of participants recounted that they read it in newspapers, on the Internet or learned it from health facilities. All participants claimed they learned about TB in their home countries, but none claimed to have received any TB-related information in Singapore.

*“No, no one has told us anything about this after we came to Singapore. We don’t know much about it either.” (M11-B)*.

*“In Singapore, no one told me anything, so I don’t know. In China, I know they say it can be treated.” (M21-C)*.

Additionally, a small number of participants attributed the lack of knowledge and awareness to their busy work schedule and the absence of discussion in the migrant community, as depicted by this participant:

*I: “[…] Do they have any discussion or knowledge dissemination on TB from your company or elsewhere?”*

R: “No, there’s no knowledge dissemination as such.” (M18-B)

### Main theme 2. Contextual knowledge and perception of associated policies related to TB in Singapore: low awareness among migrant workers

Participants’ accounts depicted a lack of information sources in Singapore, especially on issues related to healthcare, including TB. In this section, participants’ knowledge and perception of TB-related policies and services in Singapore are further categorised into two sub-themes: “Pre-entry medical screening”, and “Perceived availability of TB-related services”.

### Subtheme 2.1: Pre-entry medical screenings

All participants understood that it was essential to pass the medical examination (including screening for TB) in their home countries prior to obtaining a work permit in Singapore. The participants explained the procedures that needed to be taken before receiving work permits and working in Singapore:

*“When we enter Singapore, we had to go through medical check-up. If we have Tuberculosis, Hepatitis or something, we cannot work in Singapore. […] If you have these diseases, you will have to return to your home country.” (M01-B)*.

*“I’ve never heard of migrant workers having TB in Singapore. […] if you have hepatitis or other contagious diseases when you have your physical examination in China, they won’t approve your permit. Therefore, if you have this disease (TB), Singapore won’t let you stay. They can only treat you until you are no longer contagious, then send you back to China.” (M15-C)*.

However, participants’ accounts reflected a lack of understanding of the purpose of each medical examination. Almost all did not know what conditions the examination screened for. Many remembered having to go for a chest X-Ray but could not elaborate on the reason for it. Most shared that no explanation was ever provided to them, which is evidenced by this participant:

*“No screening is conducted for tuberculosis, but they do medical check-up, they check blood, urine, etc.” (M12-B)*.

### Subtheme 2.2: Perceived accessibility of services and cost of treatment

Participants’ narratives showed low awareness of service availability and health financing related to TB in Singapore. Apart from the compulsory physical examination for obtaining work permits, most participants admitted that they had never heard of other TB-related policies in Singapore. Several participants mentioned that immediate quarantine was required for confirmed TB patients in Singapore. In terms of treatment, most Chinese participants shared that they could get free TB treatment in China but were unsure if the same policy would apply in Singapore. Almost half of them were unsure if health services were available for migrants with TB and where they could receive TB services in Singapore. Others were confident that they could approach their company’s panel clinics or primary and tertiary care centres for help if needed.

*“Yes, in the usual way, I will have to visit the company doctor first and then the doctor will be able to tell me where to go next.” (M08-B)*.

The remaining suggested that they might approach HealthServe for advice should they suspect themselves of having TB.

Most participants were not sure about the estimated cost for TB treatment in Singapore and who would pay for the treatment. Some participants were confident that treatment costs would be cheaper in their home countries than in Singapore, as indicated by the following Chinese participant:

*“Because in China, if you have pulmonary TB, you’ll get free treatment.” (M21-C)*.

In terms of medical bills, several participants believed that employers were responsible for covering the treatment cost, as stated by one participant:

*“Treatment of tuberculosis is supposed to be provided by the company (M03-B)”.*

### Main theme 3. Attitude to towards TB: Motivation to seek treatment is underpinned by ability to continue working

Participant’s attitudes towards TB are illustrated through two sub-themes: “fatalism, lifestyle, and perceived severity” and “willingness to seek care”.

### Subtheme 3.1: Fatalism, lifestyle, and perceived severity of TB

Most participants perceived that even though the chance was low, they had no control over contracting TB. As shown in the following quotes, some Bangladeshi participants viewed this as Allah’s will. Other Bangladeshi participants suggested that there were environmental factors that were beyond their control. On the other hand, several Chinese participants believed that they would not get TB due to their healthy lifestyle and strong immunity.

*“If Allah wishes, I don’t think I will ever face anything like that. But still, one can never say what God has written for us.”* *(M07-B)*

*“[…] Friends around me don’t have that disease, one is that the infection chance is very little, right, plus my own self, like nowadays the health problem, sanitary condition are all getting better, have shower everyday […] it shouldn’t happen.” (M26-C)*.

In terms of severity, most participants believed that TB is a severe and fatal disease. A small number of participants even perceived TB to be more severe than cancer and AIDS, as illustrated in the following quote:

*“Cancer kills the one who is affected, but when TB happens to one person, it kills everybody.” (M06-B).*

### Subtheme 3.2: Willingness to seek care

Sustaining the ability to work and provide for their families were cited by most participants to be their highest life priority. As a result, seeking treatment and maintaining physical health were perceived as significant. Most participants, regardless of their nationalities, reiterated that they would immediately seek healthcare or treatment if they suspected themselves to be infected with TB. Should TB treatment be prescribed, participants stated that they would strictly follow doctors’ prescriptions and advice in order to recover. The following narrative was typical among the participants:

*“First is my physical health. If that is well, everything else follows. If my own physical health is well, nothing will happen to my family. […] So, my first action will be to take advice from a doctor.” (M09-B)*.

### Main theme 4. Stigma: mixed perception of how society views TB patients

There were mixed responses on TB-related stigma from the participants, but many highlighted that they did not have any negative perceptions of TB patients and could view the situation objectively by encouraging TB patients to seek and follow appropriate treatment. For example, a Chinese participant responded positively:

*“In normal circumstances, I would still want to befriend with him. I won’t treat him differently, such as despising him or discriminating him.” (M21-C)*.

A number of participants reasoned that the availability of effective treatment and increasing awareness among the public helped reduce the stigma toward TB patients:

*“People know that there are medicines for tuberculosis, and it can be recovered. So, that’s why people don’t get worried these days. And if they get to know about this, they don’t take it negatively now.” (M03-B)*.

Meanwhile, some participants admitted that negativity around TB patients still existed in society, potentially due to TB’s contagiousness.

*“It is seen negatively because […] it is contagious. […] They keep themselves away. Plates and utensils are kept separate.” (M18-B)*.

## Discussion

In this qualitative study, we have identified gaps in migrant workers’ TB knowledge, their attitude towards the disease and their perception of the availability of TB-related services. This is despite Singapore’s efforts to curb community spread of TB and its proactive Singapore Tuberculosis Elimination Programme (STEP) [20]. We found that misconceptions about TB were prevalent among migrant workers in Singapore. The migrant worker community, in general, did not harbour negative views toward existing TB patients. Fearing that they would lose their ability to work and support their families, the migrant workers were willing to seek health services if they had symptoms suggestive of TB. However, one of the barriers to their health-seeking identified from this study was the low awareness of the health financing and TB-related services available among migrant workers as insufficient information on TB was provided to migrant workers after they arrived in Singapore and they were unsure of where such information could be accessed.

Consistent with previous studies in migrant populations, our research suggested that participants often had inaccurate and inadequate knowledge of TB, its causes and prevention measures [21, 22]. Particularly, we found that participants tended to inappropriately generalise their knowledge about a few common infectious diseases, such as influenza, to TB. Furthermore, contrary to several earlier studies, most participants believed that TB is curable, but they had little knowledge about the mode and duration of TB treatment [21, 23]. These misconceptions could be attributed to limited access to TB-related information, both in their host and home countries. Our study showed that migrants mainly learned about TB through witnessing infected family and friends rather than from systematic and reliable sources which are lacking in their home and host countries. This finding is corroborated by a number of previous studies [22, 24].

Our study participants reported being willing to seek immediate medical attention when they had developed symptoms suggestive of TB. This finding is contradictory to other studies in which migrants were found to be reluctant to seek TB care due to various reasons such as fear of being stigmatised and poor access to health services [25–27]. This inconsistency could be due to contextual differences in study settings. Migrants in Singapore are mostly economic migrants and being able to send remittance home is their ultimate purpose. Physical health ensures their ability to continue working and supporting their families back home [28]. TB treatment, including medication and, in rare cases, hospitalisation, is free and the cost will be fully covered by the Direct Observation Therapy (DOT) program. Consultation fees and fees for clinical investigation are not covered and, in theory, should be paid by employers. However, there have been instances whereby employers failed or refused to cover the costs, or migrant workers harboured the perception that employers would not cover the costs of medical treatments despite the mandatory insurance coverage [29, 30]. Such instances might hinder migrants from seeking help immediately.

On the other hand, stigma may have a significant impact on an individual’s willingness to seek diagnosis and treatment for TB [31–33]. However, we have seen a generally neutral view towards TB patients among the migrant workers, as participants expressed their willingness to support and encourage their co-workers who had symptoms suggestive of TB to seek treatment. This is contrary to earlier studies in which stigma and avoidance toward TB patients were often observed among study populations [34–36]. This was seen in migrant workers in China, where these workers would fail to provide the healthcare providers with their contact details for monitoring and follow-up for fear of losing their livelihood if others around them found that they had developed active TB [37]. We reason that the increasing number of people among the migrant worker population with the belief that TB is curable has partially contributed to the reduced stigma. Another contributing factor could be that the level of TB-related knowledge among our study population is too low to let them make assumptions about how they would react toward TB patients.

Another important finding of our study is that the awareness of the local TB policies was low among the migrant workers in Singapore. Apart from the compulsory medical examination, the participants were not sure if they could get free TB treatment or where to seek TB-related services. Furthermore, consistent with previous studies on the health-seeking behaviour of migrant workers in Singapore, we also noted in our study that migrant workers were insufficiently aware of their rights to healthcare [10, 11]. For example, some workers were asked to do medical examination procedures without being comprehensively explained the purpose of the procedure by doctors or employers. Additionally, some of our respondents were unclear about the coverage of their health insurance and the responsibilities of employers, which might result in lower uptake of TB-related services for those who might be suspected of contracting it. Such scenarios also play out among migrant workers in South Korea due to low health literacy and thus, poor understanding of health conditions, which is aggravated by a language barrier that creates a barrier to health-seeking behaviour [38]. However, for our study, the lack of awareness can be explained by three factors. First, the compulsory TB screening prior to the issuance of work permits has successfully kept the TB incidence low among the migrant worker population. Therefore, as reported by the participants, discussions on TB-related topics among the migrant workers are scarce. This could be due to their lower general awareness to developing the disease. Second, while Singapore has been sustaining a comparatively low TB incidence after the successful implementation of the Singapore TB Elimination Program in the 2000s, health priorities shifted to non-communicable diseases when the country declared War on Diabetes in 2016, and later to COVID-19 in 2020 after the pandemic started. With little augmentation to TB campaigns and educational interventions, migrant workers have limited access and exposure to TB-related information after they arrive in Singapore. Finally, employers are required to provide medical insurance coverage of over S$ 15,000 a year to migrant workers holding Work permits or S passes by law [39]. However, information on insurance coverage or the health services that migrant workers are entitled to is often not provided or explained by employers. This is evidenced by a previous study in which 72.4% of the study participants reported that they had not, or were not sure if they had, received information about their medical insurance [10].

Therefore, there is an impetus to provide information on TB and its related services in an accessible manner for migrant workers. This can come in the form of regular advocacy in migrant worker dormitories, opportunistic education campaigns when migrants present themselves in healthcare settings for other illnesses, and co-creating culturally sensitive TB programmes to raise overall awareness in this vulnerable group.

The COVID-19 pandemic brought to the fore the fissures between the migrant workers and the general population. In Singapore, the huge number of cases of COVID-19 in foreign worker dormitories has led to renewed commitments towards improving the health of this vulnerable group. In 2020, during the midst of the pandemic, migrant workers with both TB and COVID-19 surfaced in these dormitories, which were highly congested [40, 41]. This illuminates the ongoing inequities facing this population, not only regarding TB and other healthcare services but also in the broader context, the discrepancies in providing the basic necessities centred on human rights for these migrant workers. However, the COVID-19 pandemic offers a window of opportunity to change that. The Singapore government has since rapidly implemented a new policy that improved healthcare access for migrant workers at the primary care level. The Primary Care Plan (PCP) which mandates all employers to subscribe to a medical scheme that offers essential services at primary care clinics in close proximity to migrant workers’ place of employment or residence in mid-2022, aims to narrow the inequity gap [42]. This is a step in the right direction, but access to these services and if they include TB-relevant services in an accessible manner remains to be seen.

### Recommendations

Even though we have seen willingness to seek TB care among migrant workers, lack of accurate TB knowledge and low awareness of their healthcare rights will cause delays in seeking care and receiving of TB-related services [11, 21, 31, 32]. Based on our findings and existing literature, we have developed a list of policy recommendations to improve access to TB-related services and eliminate the spread of TB in line with the UN HLM declaration for stopping TB.

Firstly, a commitment to maintain rapid contact tracing for TB cases and close contacts can be reaffirmed and best practices on contact tracing strategies used in the COVID-19 pandemic can be assimilated into current protocols [43]. This can also include stronger sentinel surveillance in the community to identify and mitigate the spread early. Secondly, the health system needs to make operations and importance of TB programmes impervious to shocks to the system so that they continue to run regardless of ongoing health emergencies [44]. Third, bidirectional screening or integration of other health services, such as non-communicable diseases at different points of care, can be explored as a comprehensive strategy by performing opportunistic screenings for TB when migrant workers present themselves with other conditions [14]. Fourth, the use of digital health tools through virtual observation therapy in place of direct physical observation can be explored to save resources for both providers and migrant workers [45]. Fifth, accurate, reliable information on the disease and factors pertaining to treatment accessibility in terms of financing need to be offered to the migrant workers. Palatability for such educational or advocacy programmes can be co-designed with the target population to overcome cultural or language barriers to maximise effectiveness. Lastly, the health system needs to support person-centred care by not only providing medical help but also addressing other non-medical issues in a human-rights-centred approach. Going forward, TB policies need to be just, fair and equitable such that migrant workers who have activated TB are not penalised by employers when they seek medical treatments. A contextualised framework to guide policymakers in crafting evidence-backed policies to promote the uptake of TB-relevant services for migrant workers should also be explored.

### Strengths and limitations

To our knowledge, this is the first qualitative study in Singapore that explored low-skilled migrant workers’ knowledge, attitudes and perceptions of TB. The findings provide a baseline and reference for future relevant research. In addition, participants from Bangladesh and China were included and interviewed in their native languages to ensure easier expression of their thoughts. Participants from different cultural backgrounds provided a breadth of views and perspectives. Interviewing participants in their native languages allowed us to build rapport and reduce misunderstanding or misinterpretation of their responses. The study was also strengthened by adopting a semi-structured interview guide during data collection. This method allowed topics beyond the topic guide to emerge, contributing to a comprehensive understanding of how participants perceived their world. However, the study team acknowledges that since only Bangladeshi and Chinese workers were recruited, further studies should include workers from other countries to improve the generalizability of our findings to the broader migrant worker population in Singapore. Additionally, the treatment of TB is a relatively long-drawn process, with interventions such as Direct Observation Therapy (DOT) spanning over months. Thus, future research should also account for the entire continuum of TB care to encompass elements such as loss to follow-up during the diagnostic period, pre-treatment and treatment phases.

We recruited our participants from an NGO clinic which served migrant workers. This might have contributed to sampling bias because those who attended the clinic session might have more positive health-seeking behaviours. In addition, almost all Chinese participants were recruited from the drop-in centre of the NGO clinic. The drop-in centre was meant for workers who experienced occupational injuries and were waiting for worker’s compensation or repatriation results. These experiences could have distorted their views on health and healthcare. Interviewing employers on their views on managing their workers who develop active TB will also be needed going forward, as employers usually have the final say regarding whether a worker remains in Singapore or gets repatriated post-diagnosis.

## Conclusion

Findings from our study suggest that misconceptions about TB symptoms, causes and treatment are prevalent among low-skilled migrant workers in Singapore. Even though migrant workers are willing to seek health services when they have symptoms suggestive of TB, low awareness of local TB policies, health insurance coverage and medical services available for migrant workers may prevent them from seeking care, thus leading to delays in TB diagnosis and treatment. Therefore, education programs that provide reliable TB knowledge and interventions that empower migrant workers by improving awareness of their healthcare rights are essential and can be conducted in the migrant worker communities in the future. Crucially, the COVID-19 pandemic has illuminated the health disparities resulting in large clusters of cases emerging among migrant populations, thus providing an ephemeral opportunity to push for more equitable healthcare services for vulnerable groups writ large [46].

### Electronic supplementary material

Below is the link to the electronic supplementary material.

Supplementary Material 1

## Data Availability

The datasets generated and/or analysed during the current study are available from the corresponding author on reasonable request.

## Footnotes

1 Work permit holders who mainly work as domestic helpers or construction workers.

## Abbreviations

LBTI: Latent Tuberculosis Infection
LMICs: Low- and middle-income countries
NGO: Non-Governmental Organisation
PCP: Primary Care Plan
STEP: Singapore Tuberculosis Elimination Programme
TB: Tuberculosis
UN HLM: United Nations High-Level Meeting
WHO: World Health Organization

## References

[1] Global tuberculosis report. 2018 [Internet]. [cited 2023 Apr 8]. Available from: https://apps.who.int/iris/handle/10665/274453.

[2] MOH | Communicable Diseases Surveillance in. Singapore 2017 [Internet]. [cited 2023 Apr 8]. Available from: https://www.moh.gov.sg/resources-statistics/reports/communicable-diseases-surveillance-in-singapore-2017.

[3] Ministry of Manpower Singapore [Internet]. [cited 2023 Apr 8]. Foreign workforce numbers. Available from: https://www.mom.gov.sg/documents-and-publications/foreign-workforce-numbers.

[4] Latent tuberculosis infection. : updated and consolidated guidelines for programmatic management [Internet]. [cited 2023 Apr 8]. Available from: https://apps.who.int/iris/handle/10665/260233. 30277688

[5] Houben RMGJ, Dodd PJ (2016). The global burden of latent tuberculosis infection: a re-estimation using Mathematical Modelling. PLoS Med.

[6] Getahun H, Matteelli A, Abubakar I, Aziz MA, Baddeley A, Barreira D (2015). Management of latent Mycobacterium tuberculosis infection: WHO guidelines for low tuberculosis burden countries. Eur Respir J.

[7] Ministry of Manpower Singapore [Internet]. [cited 2023 Apr 8]. Medical examination for migrant worker. Available from: https://www.mom.gov.sg/passes-and-permits/work-permit-for-foreign-worker/sector-specific-rules/medical-examination.

[8] Western Cape Department of Health in collaboration with the National Institute for Communicable Diseases, South Africa (2021). Risk factors for Coronavirus Disease 2019 (COVID-19) death in a Population Cohort Study from the western Cape Province, South Africa. Clin Infect Dis off Publ Infect Dis Soc Am.

[9] Kandula NR, Kersey M, Lurie N (2004). Assuring the health of immigrants: what the leading health indicators tell us. Annu Rev Public Health.

[10] Ang JW, Chia C, Koh CJ, Chua BWB, Narayanaswamy S, Wijaya L (2017). Healthcare-seeking behaviour, barriers and mental health of non-domestic migrant workers in Singapore. BMJ Glob Health.

[11] Lee W, Neo A, Tan S, Cook AR, Wong ML, Tan J (2014). Health-seeking behaviour of male foreign migrant workers living in a dormitory in Singapore. BMC Health Serv Res.

[12] Sadarangani SP, Lim PL, Vasoo S. Infectious diseases and migrant worker health in Singapore: a receiving country’s perspective. J Travel Med. 2017;24(4). 10.1093/jtm/tax014 28426114

[13] Tomás BA, Pell C, Cavanillas AB, Solvas JG, Pool R, Roura M (2013). Tuberculosis in migrant populations. A systematic review of the qualitative literature. PLoS ONE.

[14] Foo CD, Shrestha P, Wang L, Du Q, Basteiro ALG, Abdullah AS (2022). Integrating tuberculosis and noncommunicable diseases care in low- and middle-income countries (LMICs): a systematic review. PLOS Med.

[15] Velleca M, Malekinejad M, Miller C, Abascal Miguel L, Reeves H, Hopewell P (2021). The yield of tuberculosis contact investigation in low- and middle-income settings: a systematic review and meta-analysis. BMC Infect Dis.

[16] Jarde A, Romano E, Afaq S, Elsony A, Lin Y, Huque R (2022). Prevalence and risks of tuberculosis multimorbidity in low-income and middle-income countries: a meta-review. BMJ Open.

[17] TB Online - Four. years down, one to go: The road to the 2023 UN High-Level Meeting on TB [Internet]. [cited 2023 Apr 8]. Available from: https://www.tbonline.info/posts/2022/9/27/four-years-down-one-go-road-2023-un-high-level-mee/.

[18] Home - HealthServe [Internet]. [cited 2023 Apr 8]. Available from: https://www.healthserve.org.sg/.

[19] Peterson BL. Thematic Analysis/Interpretive Thematic Analysis. In: The International Encyclopedia of Communication Research Methods [Internet]. John Wiley & Sons, Ltd; 2017 [cited 2023 Apr 8]. p. 1–9. Available from: https://onlinelibrary.wiley.com/doi/abs/10.1002/9781118901731.iecrm0249.

[20] Tam G, Lai SW (2019). Is Singapore on track to eliminate tuberculosis by 2030? A policy case study. SAGE Open Med.

[21] Wieland ML, Weis JA, Yawn BP, Sullivan SM, Millington KL, Smith CM (2012). Perceptions of tuberculosis among immigrants and refugees at an adult education center: a community-based participatory research approach. J Immigr Minor Health.

[22] Gilpin C, de Colombani P, Hasanova S, Sirodjiddinova U (2011). Exploring TB-Related knowledge, attitude, Behaviour, and practice among migrant workers in Tajikistan. Tuberc Res Treat.

[23] Horner J (2016). From exceptional to liminal subjects: reconciling tensions in the Politics of Tuberculosis and Migration. J Bioethical Inq.

[24] Vukovic DS, Nagorni-Obradovic LM (2011). Knowledge and awareness of tuberculosis among Roma population in Belgrade: a qualitative study. BMC Infect Dis.

[25] Hayward S, Harding RM, McShane H, Tanner R (2018). Factors influencing the higher incidence of tuberculosis among migrants and ethnic minorities in the UK. F1000Research.

[26] Huffman SA, Veen J, Hennink MM, McFarland DA (2012). Exploitation, vulnerability to tuberculosis and access to treatment among Uzbek labor migrants in Kazakhstan. Soc Sci Med 1982.

[27] Sagbakken M, Bjune GA, Frich JC (2010). Experiences of being diagnosed with tuberculosis among immigrants in Norway — factors associated with diagnostic delay: a qualitative study. Scand J Public Health.

[28] Tam WJ, Goh WL, Chua J, Legido-Quigley H (2017). 健康是本钱 - health is my capital: a qualitative study of access to healthcare by chinese migrants in Singapore. Int J Equity Health.

[29] Ministry of Manpower Singapore [Internet]. 2015 [cited 2023 Apr 8]. Employers must foot foreign workers’ medical bills. Available from: https://www.mom.gov.sg/newsroom/press-replies/2015/1222-employers-foot-foreign-workers-med-bills.

[30] Ang JW, Koh CJ, Chua BW, Narayanaswamy S, Wijaya L, Chan LG (2020). Are migrant workers in Singapore receiving adequate healthcare? A survey of doctors working in public tertiary healthcare institutions. Singap Med J.

[31] Poss JE (1998). The meanings of tuberculosis for mexican migrant farmworkers in the United States. Soc Sci Med 1982.

[32] Xu B, Fochsen G, Xiu Y, Thorson A, Kemp JR, Jiang QW (2004). Perceptions and experiences of health care seeking and access to TB care–a qualitative study in rural Jiangsu Province, China. Health Policy Amst Neth.

[33] West EL, Gadkowski LB, Ostbye T, Piedrahita C, Stout JE (2008). Tuberculosis knowledge, attitudes, and beliefs among North Carolinians at increased risk of infection. N C Med J.

[34] Wieland ML, Weis JA, Olney MW, Alemán M, Sullivan S, Millington K (2011). Screening for tuberculosis at an Adult Education Center: results of a community-based participatory process. Am J Public Health.

[35] Marks SM, Deluca N, Walton W (2008). Knowledge, attitudes and risk perceptions about tuberculosis: US National Health interview survey. Int J Tuberc Lung Dis off J Int Union Tuberc Lung Dis.

[36] Møller H (2010). Tuberculosis and colonialism: current tales about tuberculosis and colonialism in Nunavut. Int J Indig Health.

[37] Bele S, Jiang W, Lu H, You H, Fan H, Huang L (2014). Population Aging and migrant workers: bottlenecks in Tuberculosis Control in Rural China. PLoS ONE.

[38] Novrinda H, Han DH (2022). Oral health inequality among indonesian workers in South Korea: role of health insurance and discrimination factors. BMC Oral Health.

[39] Ministry of Manpower Singapore [Internet]. [cited 2023 Apr 8]. Employment of Foreign Manpower Act. Available from: https://www.mom.gov.sg/legislation/employment-of-foreign-manpower-act.

[40] Tham SM, Lim WY, Lee CK, Loh J, Premkumar A, Yan B (2020). Four patients with COVID-19 and tuberculosis, Singapore, April-May 2020. Emerg Infect Dis.

[41] Koh D (2020). Migrant workers and COVID-19. Occup Environ Med.

[42] Ministry of Manpower Singapore [Internet]. 2022 [cited 2023 Apr 8]. Mandatory Primary Care Plan To Cover Outpatient Costs For CMP Or Dormitory-Residing Work Permit And S Pass Holders. Available from: https://www.mom.gov.sg/newsroom/press-releases/2022/0219-mandatory-purchase-of-primary-care-plans-for-certain-migrant-workers.

[43] U.S. Agency for International Development [Internet]. 2022 [cited 2023 Apr 8]. Integrating TB Screening Into COVID-19 Screening Clinics In Burma | Basic Page. Available from: https://www.usaid.gov/global-health/health-areas/tuberculosis/resources/news-and-updates/global-accelerator-end-tb/stories/tb-screening-covid-19-burma.

[44] The clock is ticking for. tuberculosis care and prevention [Internet]. [cited 2023 Apr 8]. Available from: https://www.who.int/singapore/news/commentaries/the-clock-is-ticking-for-tuberculosis-care-and-prevention.

[45] Ngwatu BK, Nsengiyumva NP, Oxlade O, Mappin-Kasirer B, Nguyen NL, Jaramillo E (2018). The impact of digital health technologies on tuberculosis treatment: a systematic review. Eur Respir J.

[46] Goh OQ, Islam AM, Lim JCW, Chow WC (2020). Towards health market systems changes for migrant workers based on the COVID-19 experience in Singapore. BMJ Glob Health.

