# Supplementary material for: A qualitative exploration of factors that influence the uptake of tuberculosis services by low-skilled migrant workers in Singapore
Source: BMC Health Serv Res. 2023 Sep 2;23:943. doi: 10.1186/s12913-023-09938-y (PMC10475191; doi:10.1186/s12913-023-09938-y)
Supplement: Supplementary file 1 — Supplementary Material 1 [file 12913_2023_9938_MOESM1_ESM.doc]

**Standards for Reporting Qualitative Research (SRQR)**

O’Brien B.C., Harris, I.B., Beckman, T.J., Reed, D.A., & Cook, D.A. (2014). Standards for reporting qualitative research: a synthesis of recommendations. *Academic Medicine, 89(9)*, 1245-1251.

| **No. Topic** | **Item** |
| --- | --- |
| **Title and abstract** |  |
| S1 Title | A qualitative exploration of factors influencing the uptake of tuberculosis services by low-skilled migrant workers in Singapore |
| S2 Abstract | **Introduction**  Singapore relies heavily on migrant workers to build its country and harbours a relatively large population of these workers. Importantly, tuberculosis (TB) remains a pernicious treat to the heath of these workers and in line with the United Nations High-Level Meeting in 2023. This paper aims to uncover the qualitative discourse facing migrant workers’ uptake of TB services and provide policy recommendations to enable more equitable access to TB services for this population.  **Methods**  In-depth interviews were carried out with the migrant worker population recruited from a non-governmental organisation in Singapore that serves migrant workers through the provision of primary health services, counselling, and social assistance. Interviews stopped once thematic saturation was achieved and no new themes and subthemes were found.  **Results**  A total of 29 participants were interviewed, including 16 Bangladeshis and 13 Chinese, aged between 22 and 54 years old, who all worked in the construction sector. Four key themes emerged. They are 1) General TB knowledge: Misconceptions are prevalent, where we found that participants were aware of the disease but did not possess a clear understanding of its pathophysiology and associated health effects, 2) Contextual knowledge and perception of associated policies related to TB in Singapore: low awareness among migrant workers as participants’ accounts depicted a lack of information sources in Singapore especially on issues related to healthcare including TB, 3) Attitude to towards TB: Motivation to seek treatment is underpinned by ability to continue working and 4) Stigma: mixed perception of how society views TB patients. The gaps identified in migrant workers’ TB knowledge, their attitude towards the disease and their perception of the availability of TB-related services is despite Singapore’s efforts to curb community spread of TB and its proactive initiatives to reduce the prevalence.  **Conclusion**  Our study illuminates the various aspects that policymakers need to home in on to ensure this vulnerable group is sufficiently supported and equitably cared for if they develop active TB during their stay in Singapore as they continue to the nation’s economy. Leveraging the COVID-19 pandemic as a window to improve overall healthcare access can be a starting point. |
| **Introduction** |  |
| S3 Problem formulation | TB remains a pertinent issue in Singapore, a high-income country that remains heavily reliant on low-skilled migrant workers, most of whom hail from countries with relatively high TB burdens. Although these migrant workers undergo stringent health checkups before entry into Singapore, it is pre-emptive and pragmatic to understand the factors influencing the uptake of TB services if they do develop active TB during their working duration in Singapore. |
| S4 Purpose or research question | This research aims to address this gap by offering a qualitative discourse on migrant workers' uptake of TB-related services in Singapore. |
| **Methods** |  |
| S5 Qualitative approach and research paradigm | A qualitative approach to the research question was employed by recruiting prospective participants for semi-structured, in-depth interviews. The audio tapes were transcribed verbatim and thematic analysis was performed to allow the key themes pertaining to TB and uptake of TB-relevant services by low-skilled migrant workers to emerge. This process of parallel participant recruitment, interviewing, transcribing and analysis was performed until data saturation was reached. Whenever disagreements surface, the expanded team will iteratively discuss to co-create a theme with everyone’s consensus. |
| S6 Researcher characteristics and reflexivity | The researchers are all public health trained and have experience in qualitative research. All team members provided feedback at every stage of data collection, consolidation and analysis to co-create the themes that fit the research question. |
| S7 Context | The participants were recruited from an NGO health service centre (Health Serve), where the most vulnerable populations of low-skilled migrant workers would appear to seek help for healthcare services. Also, a qualitative approach, i.e., semi-structured in-depth interviews, was the most appropriate for this research as we wanted to explore the factors influencing the uptake of TB services and perspectives around the disease, which is best understood in a semi-open interview format with correct probing questions. |
| S8 Sampling strategy | Prospective participants were recruited from an NGO which offered healthcare services to low-skilled migrant workers. The researchers would approach the workers, elaborate on the study, and obtain their informed written consent before the interview. The sampling was purposive, as only low-skilled migrant workers were recruited. However, only two main nationalities were recruited, which might impact our findings’ generalizability. We have included this in our limitations section. |
| S9 Ethical issues pertaining to human subjects | We have obtained full institutional board approval from the National University of Singapore Institutional Review Board (NUS-IRB: S-18-197). All participants were explained the study and written informed consent was obtained before commencing the interviews. All audio tapes and subsequent audio transcripts were de-identified as soon as possible so that the data would not be linked back to the participant. We also emphasised that not participating in our research study would not affect the health services the NGO offers them. |
| S10 Data collection methods | Data was collected through a semi-structured in-depth interview fashion and the interview took place in a quiet room that was conducive so that sensitive information could be revealed without any disruptions. The audio tapes were also de-identified at the earliest possible. |
| S11 Data collection instruments and technologies | An audio recorder and topic guide were used during the data collection process. QSR NVivo version 12 was used during the data analysis process to organise the themes and subthemes. |
| S12 Units of study | A total of 29 participants were interviewed, including 16 Bangladeshis and 13 Chinese, aged between 22 and 54 years old were recruited for this study. All participants were recruited through the NGO and were low-skilled migrant workers who were working in Singapore. |
| S13 Data processing | The audio files that were obtained from the semi-structured in-depth interviews were transcribed verbatim. The audio transcripts were de-identified at the earliest and stored in an encrypted folder that was accessible only to the research team. The audio transcripts were only used for data analysis for the purposes of this qualitative research study. All quotes used in this paper are de-identified so that the quotes will not be linked to the participants. |
| S14 Data analysis | The research team proofread all audio transcripts before data analysis to ensure that the transcription and translation were done literally without losing accurate meanings. QSR NVivo 12 software was used to manage and organise the data. Two authors first coded half of the transcripts each, line-by-line and inductively. The interviews were approached through an interpretivist lens to focus on participants’ experiences, perceptions and how they understand and reason with the topic discussed. After the initial coding was completed, the two authors compared the two sets of codes to ensure inter-coder reliability and allow data triangulation. The coders were engaged in regular discussions to identify emerging themes and seek out deviant cases. In the meantime, recruitment and interviews continued in parallel, followed by audio transcription and thematic analysis. These collection, consolidation and analysis steps only stopped when thematic saturation was reached and no new themes had emerged. Disagreements were resolved by discussing and consulting with a third researcher who provided neutral views on the data set. Eventually, a coding frame was agreed to and then used to re-code the whole set of data to ensure consistency. All authors held regular debriefings on our pre-conceived ideas and our perceptions throughout the study period to provide as much neutrality as possible. |
| S15 Techniques to enhance trustworthiness | All data collected was carefully curated by at least two authors. All disagreements in terms of thematic analysis were resolved through iterative discussions with the expanded team until a consensus was reached. Furthermore, all authors are experts in the field of healthcare access for vulnerable groups and experienced qualitative researchers. |
| **Results/Findings** |  |
| S16 Synthesis and interpretation | The findings are stated as themes and subthemes in the manuscript's results section and interpreted by the expanded research team at every stage of the analysis process. |
| S17 Links to empirical data | All audio tapes obtained from the semi-structured in-depth interviews were transcribed verbatim. The themes and subthemes were allowed to emerge from the data and linked empirically to the research question. |
| **Discussion** |  |
| S18 Integration with prior work, implications, transferability, and contribution(s) to the field | The discussion encompassed debate on the various factors that are contextualised to low-wage migrant workers in Singapore with regard to TB and uptake of TB-related services. Some of the reasons for not taking up TB-related services are apparent in other countries, and the policy recommendations are based on our results. We have also corroborated some of our findings with publications made with data sets from other countries and have included these comparisons throughout our discussion section. |
| S19 Limitations | There might be sampling bias as the participants were recruited from the same NGO that offers free healthcare services and only Chinese and Bangladeshi participants were interviewed for the study. Therefore, this might limit the generalizability of the findings to other migrant worker groups and it should examined in further work. This has been included in our limitations section. |
| **Other** |  |
| S20 Conflicts of interest | All authors declare no conflict of interest. |
| S21 Funding | This research is funded by the National Medical Research Council, Singapore, NMRC/HSRG/0056/2016. |

^a^The rationale should briefly discuss the justification for choosing that theory, approach, method, or technique rather than other options available, the assumptions and limitations implicit in those choices, and how those choices influence study conclusions and transferability. As appropriate, the rationale for several items might be discussed together.
